# The Systematic Analysis of Exercise Mechanism in Human Diseases

**DOI:** 10.1155/2022/8555020

**Published:** 2022-03-24

**Authors:** Lei Pu, Peng Sun

**Affiliations:** ^1^The School of Sports and Health of East China Normal University, Shanghai 201100, China; ^2^Key Laboratory of Adolescent Health Assessment and Exercise Intervention, Ministry of Education, East China Normal University, Shanghai 200241, China

## Abstract

**Background:**

As a part of a healthy lifestyle, exercise has been proven to be beneficial for the treatment of diseases and the prognosis of patients. Based on this, our research focuses on the impact of exercise on human health.

**Methods:**

To study and analyze the samples in the GSE18966 gene expression profile, we first performed an analysis on the differential expressed genes (DEGs) through GEO2R, and then the DEGs enrichment in Gene Ontology functions and Kyoto Encyclopedia of Genes and Genomes (KEGG) pathways through the Database for Annotation, Visualization and Integrated Discovery database was conducted. Then, we delved into the gene set enrichment in KEGG through gene set enrichment analysis. After that, we achieved the construction of the protein-protein interaction (PPI) network of DEGs based on the Search Tool for the Retrieval of Interacting Genes online database, and the hub genes were screened and identified.

**Results:**

We identified 433 upregulated DEGs and 186 downregulated DEGs from the samples before and after exercise in GSE18966. Through analysis, it was found that these DEGs-enriched pathways, such as the VEGF signaling pathway, the Wnt signaling pathway, and the insulin signaling pathway, were all involved in the regulation of various diseases. Then, GSEA analysis revealed that glycosaminoglycan biosynthesis chondroitin sulfate, type II diabetes mellitus, and basal cell carcinoma were related with exercise samples. The effects of these pathways on various diseases could be improved through exercise. Finally, 3 upregulated hub genes (VEGFA, POMC, and NRAS) and 3 downregulated hub genes (HRAS, NCOR1, and CAV1) were identified through the PPI network.

**Conclusions:**

The bioinformatic analysis of samples before and after exercise provides key pathways and genes related to exercise to regulate various diseases, which confirms that exercise has an important influence on the treatment of many diseases.

## 1. Background

As a part of the human lifestyle, exercise is an activity involving physical strength and skills, which not only enhances people's physical fitness but also enriches their social, cultural, and entertainment lives [1]. With the increasing demand for living standards, people pay more and more attention to a healthy lifestyle and actively participate in various exercises that can promote blood circulation, enhance physical fitness, and speed up metabolism [2]. In 2020, the World Health Organization issued a code of conduct on exercise and sedentary, suggesting that everyone should perform appropriate aerobic exercise every day [3]. For people with different diseases, the benefits of exercise are also slightly different. For hypertensive patients, exercise can reduce the mortality rate of cardiovascular diseases, delay the progression of the disease, improve physical function, and the quality of life [4, 5]. For cancer patients, exercise can also improve the patient's treatment prognosis. Busch and others study the effects of exercise on the treatment of fibromyalgia. Aerobic and strength exercises can improve physical function and reduce the symptoms of fibromyalgia [6]. Halabchi et al. have shown through a large amount of data that appropriate exercise can improve cardiopulmonary function, muscle strength, cognition, life quality, and so on [7]. For patients suffering from multiple sclerosis, exercise can be used as an effective and secure way of rehabilitation [8]. Although many studies have shown that exercise has a certain effect on delaying disease proliferation and improving prognosis, the regulation of molecular mechanisms in the human disease process remains to be explored.

As an intersecting biological subject, genomics mainly carries out quantitative research, collective characterization, and comparative research of different genomes. Primary methods and tools of genomics are inclusive of genetic analysis, bioinformatics, gene expression measurement, and gene function identification [9]. This method, together with transcriptomics, proteomics, and metabolomics, constitutes the omics basis of systematic biology [10]. It has been widely used in medicine, biotechnology, anthropology, and other social sciences. Microarray technology comes to appear as the Human Genome Project and is gradually implemented [11]. A transcriptome refers to the collection of all transcription products in a cell under a certain physiological condition. Through a new generation of high-throughput sequencing, transcriptome analysis can quickly and comprehensively gain nearly all the transcript sequence information of a specific tissue or organ of a certain species in a certain state and is widely used in clinical diagnosis, basic research, drug development, and other fields [12]. The comprehensive bioinformatics analysis combined with the abovementioned methods will help research diseases.

The main purpose of this study is to confirm the effect of exercise on human health. We obtained samples from the GSE18966 gene expression profile, analyzed the differential expressed genes (DEGs) in these samples, and performed Gene Ontology (GO) term and Kyoto Encyclopedia of Genes and Genomes (KEGG) pathway enrichment analyses on these DEGs with the help of the Database for Annotation, Visualization and Integrated Discovery (DAVID) database. After that, we analyzed the KEGG pathway enriched in gene sets through gene set enrichment analysis (GSEA). Finally, the protein-protein interaction (PPI) network of DEGs has been constructed in the Search Tool for the Retrieval of Interacting Genes (STRING) online database, and we identified the hub genes, and the relationship between the hub genes and exercise was studied.

## 2. Materials and Methods

### 2.1. Gene Expression Microarray Data Collection

The Gene Expression Omnibus (GEO; https://www.ncbi.nlm.nih.gov/geo) is a database that stores microarray, next-generation sequencing, and other high-throughput sequencing data. Based on this database, the gene expression profile of GSE18966 was downloaded. We saved it in txt format. We selected 10 sample data in the GSE18966 gene expression profile as the research object and divided them into 5 peripheral blood samples drawn before exercise and 5 peripheral blood samples drawn 4 hours after exercise. Based on these two sets of sample data, the next step of research could be carried out.

### 2.2. Identification of DEGs

GEO2R (https://www.ncbi.nlm.nih.gov/geo/geo2r/) is a software used for differential analysis of expression profile chips based on the GEO database, which can directly query the original series of matrix data files. Based on this tool, we analyzed the sample data in GSE18966 and set fold change (FC) >2, *P* value *<*0.05 as the screening criterion for upregulated DEGs and FC < 0.5, *P* value *<*0.05 as the screening criterion for downregulated DEGs.

### 2.3. GO and KEGG Enrichment Analysis of DEGs

As a standardized gene function classification system around the world, GO offers a dynamically updated standard vocabulary to the attributes of genes and gene products in organisms. GO consists of three parts in total, which are as follows: molecular function, cellular component, and biological process. This time, research was only conducted on the biological process (BP). Through making use of the KEGG database, we can systematically analyze link genomic information, gene functions, and functional information databases, including metabolic pathway databases, hierarchical classification databases, gene databases, and genome databases. In this study, we performed enrichment analysis on the GO function and KEGG pathway of the identified DEGs through making use of the DAVID database.

### 2.4. GSEA of Gene Set

We often evaluated the distribution trend of genes in a predefined gene set in a gene table ranked by phenotype correlation through making use of GSEA, aiming at judging their contribution to phenotype. GSEA can analyze those genes whose expression is not differentially significant but have important biological significance, which is easily missed from the GO/KEGG enrichment information; thus, there is no need to specify a threshold to screen for differential genes. With the help of GSEA software, we performed the KEGG pathway enrichment analysis on the gene set in the two sets of sample data.

### 2.5. PPI Network Construction and Hub Genes Identification Analysis

STRING is an online database for searching the relationships between known proteins. It can be used to filter and evaluate functional genomics data and to provide a more intuitive platform for annotating the structure, function, and evolution of proteins. In this research, we constructed a PPI network of upregulated DEGs and downregulated DEGs, respectively, based on the STRING database and identified the upregulated and downregulated hub genes.

## 3. Results

### 3.1. Identification of DEGs

**Figure 1:**
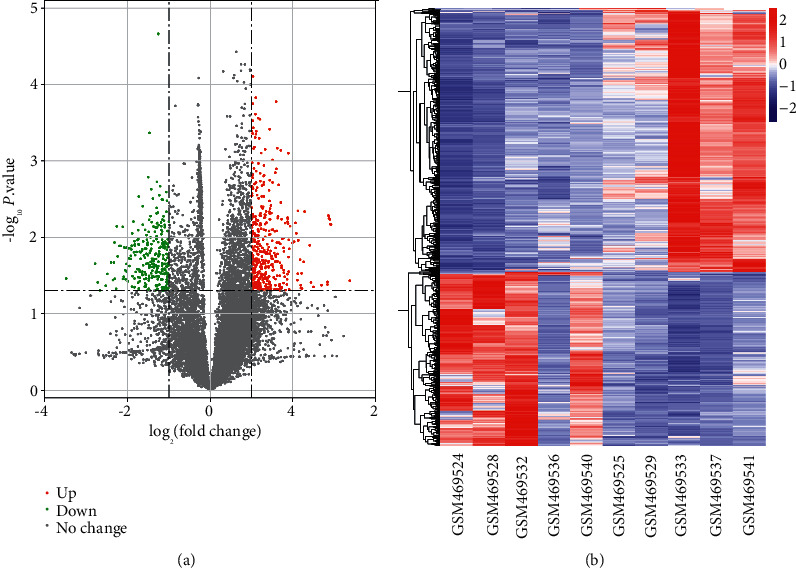
Volcano plot and heat map of DEGs. (a) Volcano plot. Green represents downregulation of DEGs, red represents upregulation of DEGs, and gray represents no significant change genes. (b) Heat map of the distribution of all DEGs in the two sets of samples.

The expression profile of GSE18966 contained 5 peripheral blood samples drawn before exercise and 5 peripheral blood samples drawn 4 hours after exercise. From these samples, we identified 719 DEGs in total, with 433 upregulated DEGs and 286 downregulated DEGs (Figure 1(a)).  Figure 1(b) heatmap showed the expression distribution of these DEGs in the 10 samples.

### 3.2. GO Function and KEGG Pathway Analysis

**Figure 2:**
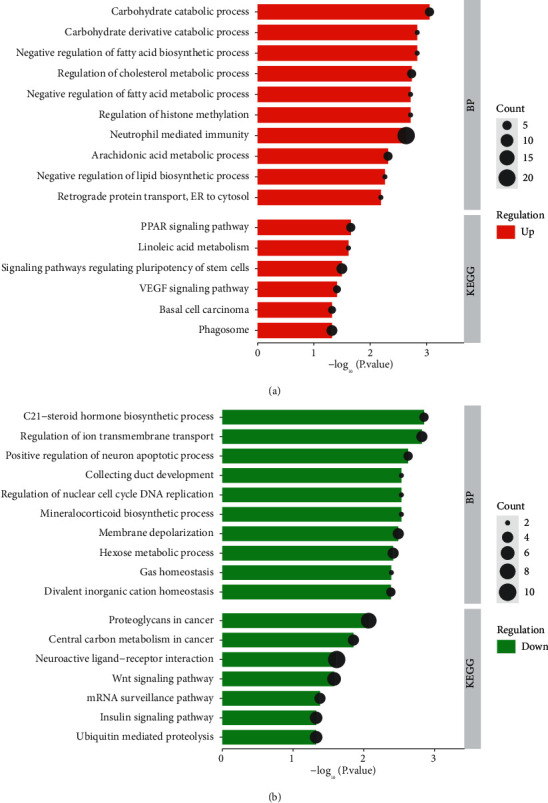
GO functional and KEGG pathway analysis of DEGs. (a) BP and KEGG enrichment analysis of upregulated DEGs. (b) BP and KEGG enrichment analysis of downregulated DEGs. The bar stands for the size of the *P* value, the longer the bar, the smaller the *P* value; the dot stands for the number of genes, the larger the dot, the more the number of genes.

Figure 2(a) shows the enrichment analysis results of upregulated DEGs in BP and KEGG. In BP, upregulated DEGs were obviously abundant in the carbohydrate catabolic process, carbohydrate derivative catabolic process, negative regulation of the fatty acid biosynthetic process, regulation of the cholesterol metabolic process, negative regulation of the fatty acid metabolic process, etc. In the KEGG pathway, the upregulated DEGs obviously enriched six pathways, namely, the PPAR signaling pathway, the VEGF signaling pathway, linoleic acid metabolism, signaling pathways regulating pluripotency of stem cells, basal cell carcinoma, and phagosome.

According to Figure 2(b), we could see that the downregulated DEGs were obviously abundant in BP in the C21-steroid hormone biosynthetic process, regulation of ion transmembrane transport, positive regulation of neuron apoptotic process, collecting duct development, regulation of nuclear cell cycle DNA replication, mineralocorticoid biosynthetic process, etc. In KEGG, these downregulated DEGs were obviously abundant in proteoglycans in cancer, central carbon metabolism in cancer, neuroactive ligand-receptor interaction, Wnt signaling pathway, mRNA surveillance pathway, etc.

### 3.3. GSEA Analysis

**Figure 3:**
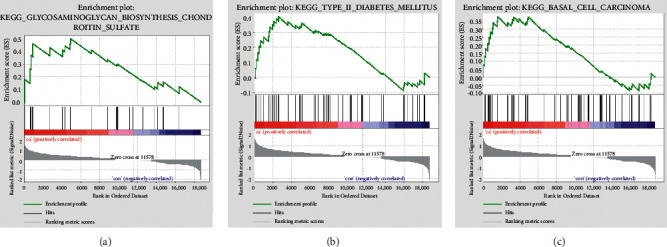
KEGG pathway related to exercise samples by GSEA analysis. (a) Glycosaminoglycan biosynthesis chondroitin sulfate. (b) Type II diabetes mellitus. (c) Basal cell carcinoma.

We analyzed the gene set in the two sets of samples through GSEA and obtained three KEGG pathways related with after-exercise gene set, namely, glycosaminoglycan biosynthesis chondroitin sulfate, type II diabetes mellitus, and basal cell carcinoma (Figures 3(a)–3(c)).

### 3.4. PPI Network Construction and Hub Genes Identification Analysis

**Figure 4:**
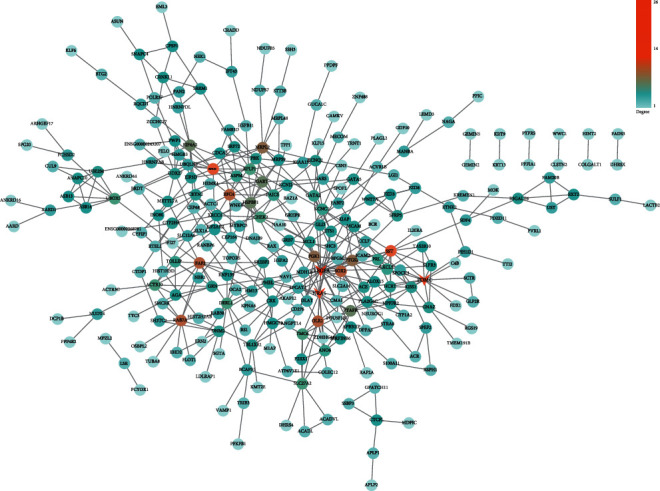
PPI network for upregulated DEGs. The nodes stand for the names of genes, and the edges stand for the interactions between genes. The color bar stands for the degree value.

**Figure 5:**
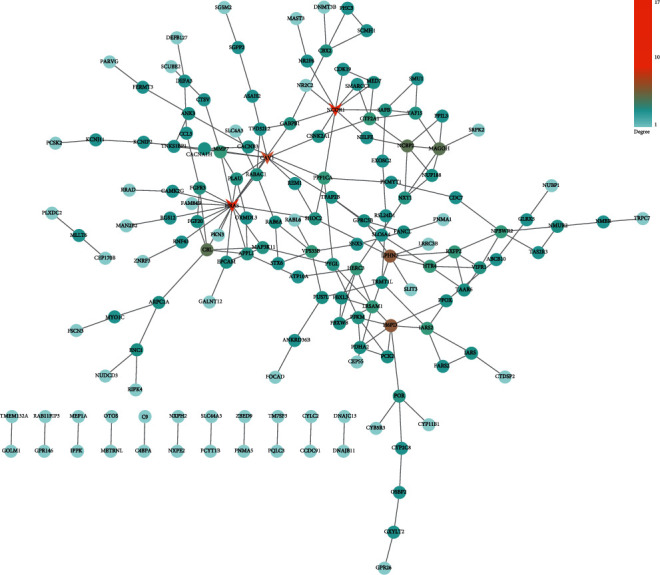
PPI network for downregulated DEGs. The nodes stand for the names of genes, and the edges stand for the interactions between genes. The color bar stands for the degree value.

**Figure 6:**
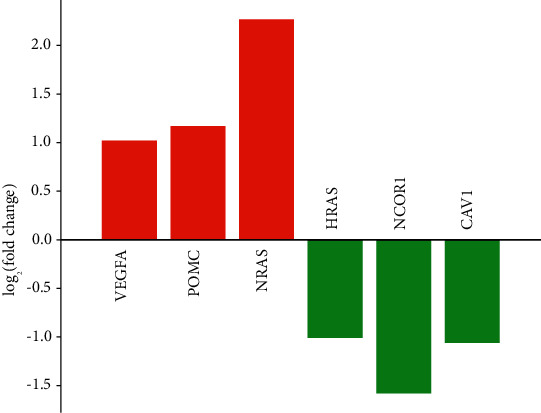
Hub gene expression analysis. Red stands for upregulated genes, and green stands for downregulated genes in exercise samples based on GSE18966.

A total of two PPI network diagrams have been established based on the STRING online network tool.  Figure 4 is a PPI network diagram of upregulated DEGs, including 449 edges and 379 nodes. Among them, the orange nodes were the genes that were significantly connected with other genes. According to the degree from the largest to the smallest, a total of three hub genes have been identified, namely, VEGFA (degree = 26), NRAS (degree = 13), and POMC (degree = 13). The PPI network graph of downregulated DEGs consisted of 194 edges and 255 nodes (Figure 5). According to the degree ranking, the first 3 genes, namely, HRAS (degree = 17), NCOR1 (degree = 10), and CAV1 (degree = 8), were selected as downregulated hub genes.  Figure 6 summarizes the fold change of hub gene expression in peripheral blood samples drawn 4 hours after exercise compared to samples before exercise.

## 4. Discussion

From the GEO database, we downloaded the GSE18966 gene expression profile and used the 5 peripheral blood samples drawn before exercise and 5 peripheral blood samples drawn 4 hours after exercise in the expression profile as the basis of the research. 719 DEGs in total were obtained, 433 upregulated DEGs and 286 downregulated DEGs included. After that, the PPI network diagrams of upregulated DEGs and downregulated DEGs were constructed, respectively. Then, 3 upregulated hub genes (VEGFA, POMC, and NRAS) and 3 downregulated hub genes (HRAS, NCOR1, and CAV1) were identified according to degree value.

Generally, peripheral blood cells can be divided into three types: white blood cells, red blood cells, and platelets [13]. Among them, white blood cells have a paramount protective function on the human body. White blood cells are deformed and pass through the capillary wall to focus on the part where the bacteria invade, surround, and swallow the bacteria when the bacteria invade the human body [14]. In addition, white blood cells can be divided into 5 types, which can be sorted according to their sizes: eosinophils, monocytes, neutrophils, basophils, and lymphocytes. Different types of white blood cells participate in defense in the human body with their different functions [15]. Peripheral blood leukocytes are a component of the innate immune system and adapt to the newly adopted immune system [16]. Abnormalities of these cells usually cause immune system disorders and are usually used as basic indicators for the clinical diagnosis of diseases and observation of curative effects. The study by Carrick and Begg shows that peripheral blood leukocytes are a key component of the immune system. Through the study of the endotoxin verification system for Gram-negative bacterial cell wall products in horses, it was found that the regulation of leukocytes will make many diseases that plague horses effectively, which provides a therapeutic target for the treatment of inflammatory diseases [16]. The research of Rokutan et al. applies the gene expression profile of peripheral blood leukocytes to the method of evaluating human stress responses, which can detect the abnormal stress response of related diseases' oncogenes [17].

We analyzed the enrichment results of upregulated DEGs and downregulated DEGs in GO and KEGG through the DAVID database. Among them, upregulated DEGs were abundant in carbohydrate catabolic process, carbohydrate derivative catabolic process, etc. in BP, and enriched in the PPAR signaling pathway, linoleic acid metabolism, the VEGF signaling pathway, etc. in KEGG. Zhao Y et al. introduced in their study that VEGF and related receptor VEGFR-2 were highly expressed in human tumors and had an important influence on the process of angiogenesis [18]. In the study carried out by Le, the role and mechanism of VEGF in diabetic animal models were explored, and the role and mechanism of VEGF signal-mediated vascular activity and neuroprotection in diabetic animal models were studied. It indicated that the VEGF signaling pathway may have important significance for the survival of Müller glia in diabetes [19]. The enriched pathways of downregulation of DEGs in KEGG, such as the Wnt signaling pathway and the insulin signaling pathway, are involved in the regulation of many diseases. Huang P et al. studied the role of Wnt signaling in hair loss, pigment disorders, wound healing, bone disease, neurodegenerative diseases, and chronic obstructive pulmonary disease, indicating that the Wnt signaling pathway had a certain potential in the treatment of diseases [20]. The insulin signaling pathway is involved in the transduction regulation in many diseases. Studies by Akhtar A et al. confirmed that the insulin signaling pathway and molecules, such as IRS, PI3K, Akt, and GSK-3*β*, have an important influence on neurodegenerative diseases and Alzheimer's disease [21]. We suggest that the abovementioned diseases related to the enriched signal pathway can achieve optimistic therapeutic effects through exercise therapy.

After that, we analyzed the KEGG pathway enriched by the gene set in the sample through GSEA and obtained three related signal pathways, namely, glycosaminoglycan biosynthesis chondroitin sulfate, type II diabetes mellitus, and basal cell carcinoma. The study by Henrotin Y et al. reported that chondroitin sulfate and glucosamine sulfate could facilitate the metabolism of joint synovial cells *in vitro*, and these cells were all related to osteoarthritis. The effect of exercise on the treatment of osteoarthritis had been confirmed by many research results [22]. Bennell and others believed that exercise should be applied to the treatment of osteoarthritis regardless of the severity of the disease and functional status [23]. Lambova analyzed different exercise training programs for different parts of osteoarthritis, which could improve neuromuscular, peripheral joints, reduce cardiovascular risk, and improve mental health [24].

Based on the STRING database, we constructed a PPI network for upregulated DEGs and downregulated DEGs and identified 3 upregulated hub genes and 3 downregulated hub genes according to the degree. The upregulated hub genes were VEGFA, NRAS, and POMC. The downregulated hub genes were HRAS, NCOR1, and CAV1. VEGFA is a member of the PDGF/VEGF growth factor family. This gene induces vascular endothelial cell migration and proliferation during angiogenesis and is involved in the expression of many known tumors. Studies have found that this gene is mainly related to POEMS syndrome and diabetic microvascular complications. Therkildsen et al. believed that NRAS could be applied to the anti-EGFR treatment of metastatic colorectal cancer [25]. Cicenas et al. study and analyze the regulatory role of NRAS in the occurrence and development of colorectal cancer and melanoma [26]. Candler et al. believe that POMC is a factor that causes satiety. After studying children, it has been found that this gene plays an important role in obesity and metabolic diseases [27]. As a member of the RAS oncogene family, HRAS is in relation to the transforming genes of mammalian sarcoma retroviruses. The study by Waters and Der confirms that this gene has a regulatory driving role in pancreatic cancer and can be used as a therapeutic target [28]. NCOR1 is a transcription inhibitor that links chromatin-modifying enzymes with gene-specific transcription factors. This gene plays a full role in energy homeostasis. Some studies have shown that this gene can be used to treat metabolic diseases such as obesity and type 2 diabetes. In addition, Kruglikov and Scherer proved through experiments that overexpression of CAV1 can cause a reduction in psoriasis inflammation and inhibit epidermal hyperplasia, which can be used as a pathological factor and a therapeutic target for psoriasis [29]. The diseases related to these genes, such as obesity, diabetes, metabolic diseases, and melanoma, can be treated by exercise. A large number of studies have confirmed that exercise can promote blood circulation, strengthen physical fitness, accelerate metabolism, control weight, lower the incidence of diseases, and reduce the incidence of cardiovascular and cerebrovascular diseases. The studies by Warner and others also show that obesity is related to improving the immune and targeted therapy of melanoma [30]. Potential factors, such as obesity, microbiota, diet, and exercise, can affect the occurrence, development, and treatment of melanoma. Therefore, it is confirmed that exercise can regulate the development of the disease by regulating the expression level of the hub gene.

In short, we analyzed the sample data in the GSE18966 gene expression profile and identified 433 upregulated DEGs and 286 downregulated DEGs after exercise. Then, through enrichment analysis, we identified that exercise was related to signaling pathways such as VEGF, Wnt, and insulin, and glycosaminoglycan biosynthesis chondroitin sulfate. After that, 6 hub genes, namely, VEGFA, NRAS, POMC, HRAS, NCOR1, and CAV1, were identified and found to be involved in various diseases. Our research has suggested that exercise can participate in the progress of disease treatment by regulating the related pathways and genes.

## Data Availability

The datasets used and/or analyzed during the current study are available from the corresponding author on reasonable request.
